# Balancing in Unicompartmental Knee Arthroplasty: Balancing in Flexion or in Extension?

**DOI:** 10.3390/jcm11226813

**Published:** 2022-11-17

**Authors:** Riccardo D’Ambrosi, Raju Vaishya, Francesco Verde

**Affiliations:** 1 IRCCS Istituto Ortopedico Galeazzi, 20161 Milan, Italy; 2 Dipartimento di Scienze Biomediche per la Salute, Università degli Studi di Milano, 20133 Milan, Italy; 3 Indraprastha Apollo Hospitals, New Delhi 110076, India; 4 IRCCS Ospedale San Raffaele, 20132 Milan, Italy

Unicompartmental knee arthroplasty (UKA) is an established procedure for the treatment of predominant single compartmental femorotibial osteoarthritis (OA) or osteonecrosis. In recent decades, the advent of the concept of minimally invasive surgery (MIS), together with the development and refinement of surgical techniques and implant design, has led to improved clinical outcomes and, consequently, renewed interest in UKA [1].

With the clear potential advantages of UKA, it is mandatory to proceed not only with quantitative but also qualitative procedures adding as much knowledge as possible. Today, it is normal clinical practice to evaluate national registries to determine the validity of a procedure or implant and evaluate patient-reported outcomes (PROMS) from the patient population [2]. This trend is to move from quantitative criteria (e.g., number of failures by Kaplan–Meier method) to qualitative criteria (e.g., PROMS) of evaluation [3]. In fact, reconstructions were made only on functional criteria earlier, while now they are also based on anatomical criteria [4].

This editorial aims to integrate the concepts of mobile and fixed bearing and flexion or extension balancing. Resurfacing is a concept related to the second generation of UKA, such as that of Allegretto or St. George [5]. At that time, the idea was to achieve true gap balancing through spreaders. However, the real limitation of the femoral resurfacing design is that it cannot allow anatomic reconstruction of the femur if the femoral deformity goes beyond 2 mm in thickness, as is encountered in most cases. To address this factual problem, Allegretto’s prosthesis has provided two sizes with an increased distal thickness (4 mm), as the design of 2 mm distal thickness does not allow correction of a 3 mm or more deformity of the femur with anatomical joint-line reproduction. In these cases, only a functional reconstruction of the space by flexion balancing is possible, accomplished by the upwards displacement of the interline [6].

Flexion balancing is characterized when the priority is to reconstruct the height and obliquity of the knee joint line. However, in extension alignment, the height and obliquity of the joint line cannot be reconstructed if the distal femur is worn. To achieve this, the cut of the tibia is measured in relation to the posterior femoral condyle in flexion because it is usually intact in almost all varus knees [7]. Once the correct laxity is found, this represents the height of the joint line. In contrast, in extension balancing, residual laxity depends exclusively on distal femoral wear; hence, it is mandatory to distalize the cut by 1 mm or more.

Alignment in extension refers to a technique that evaluates the laxity of the knee primarily and independently from the joint line and is, therefore, a functional alignment, which aims to obtain functionality in flexion and extension independently from the joint line. Current instruments are mostly oriented to extension alignment, and only a few (current Oxford) offer an option for flexion alignment.

Extension and flexion balancing are different philosophies, and the only situation where they overlap is in the case of an intact femur. Once the tibia cut is performed, the minimum thickness that reproduces the correct knee laxity is evaluated in extension. The distal femoral cut is fixed and corresponds to the thickness of the prosthetic component regardless of wear. As a result, the wear of the distal femur defines the degree of spacing elevation. To accommodate flexion laxity accordingly, the femoral component is shifted anteriorly [8,9].

Therefore, the two balancing techniques are available in the less frequent case scenario of non-wear of the distal femur. Since the percentage of non-worn distal femurs in advanced knee OA is <5%, it assumes the risk of overindication. In the rare cases where it is, the wear is not volumetrically significant and therefore does not functionally matter with respect to the surgical technique in question [10,11,12].

Instrumentations in the past were only for functional criteria, and spreaders served to equalize spaces in flexion and extension [5]. It is understood that the fundamental concept of anatomical reconstruction of the femur, as an indispensable tool to achieve anatomical joint-line reconstruction, was missing. The new instrumentation allows choosing between a functional reconstruction or an anatomical reconstruction.

In the valgus knee, the techniques overlap because the joint line is evaluated in extension since the non-worn part of the femur is usually the distal one [13]. Here, the posterior lateral femoral condyle is usually worn and cannot be used as a reference (but can be reconstructed using posterior templates). The valgus knee presents a different wear mechanism that primitively involves the tibia wearing centrally and consensually the posterior distal femur. In this case, the assessment is to be made for anatomical reconstruction, which is mostly diametrically opposite to the varus knee.
